# Circulating miRNA Profile in Inflammatory Bowel Disease Patients with Stress, Anxiety, and Depression

**DOI:** 10.3390/ijms26157321

**Published:** 2025-07-29

**Authors:** Maria Dobre, Teodora Ecaterina Manuc, Mircea Manuc, Ioan-Costin Matei, Anastasia-Maria Dobre, Andrei-Daniel Dragne, Elisabetta Maffioletti, Iulia Andreea Pelisenco, Elena Milanesi

**Affiliations:** 1 Victor Babeș National Institute of Pathology, 050096 Bucharest, Romania; maria_dobre70@yahoo.com (M.D.); elena.k.milanesi@gmail.com (E.M.); 2 Faculty of Medicine, Carol Davila University of Medicine and Pharmacy, 050474 Bucharest, Romania; teodora.manuc@gmail.com (T.E.M.); m_manuc@yahoo.com (M.M.); anastasiadobre@yahoo.com (A.-M.D.); d.andrei.daniel.work@gmail.com (A.-D.D.); 3 Fundeni Clinical Institute, 022328 Bucharest, Romania; 4 Nutrimed Clinic, 020625 Bucharest, Romania; costinmatei22@gmail.com; 5 Department of Theoretical and Applied Sciences (DiSTA), eCampus University, 22060 Novedrate, Italy; elisabetta.maffioletti@uniecampus.it; 6 Unit of Psychiaty, IRCCS Istituto Centro San Giovanni di Dio Fatebenefratelli, 25125 Brescia, Italy

**Keywords:** inflammatory bowel disease, anxiety, depression, stress, miRNAs, plasma, biomarker

## Abstract

High rates of depression and anxiety have been reported among patients with inflammatory bowel disease (IBD). The bidirectional relationship between these two conditions, with each affecting the progression of the other, leads to a reduced quality of life. The aim of this study was to identify a miRNA-based pattern that may either be unique to IBD or associated with this complex phenotype. The levels of 179 miRNAs were assessed using qRT-PCR in the plasma of individuals primarily diagnosed with recurrent depressive disorder (SAD), IBD patients (IBD), IBD patients showing symptoms of stress, anxiety, and depression (IBD + SAD), and a control group. Four miRNAs were found to be specifically associated with IBD and more than 20 miRNAs with SAD. Notably, the levels of five miRNAs (miR-223-3p, miR-1260a, miR-320d, miR-423-5p, and miR-486-5p) differed in all the comparisons. miR-342-3p and miR-125a-5p were identified as possible biomarkers able to discriminate between IBD and IBD + SAD. The identification of this pattern of miRNA specific to IBD + SAD could be useful for monitoring disease activity and progression in IBD patients struggling with psychiatric symptoms, which can negatively impact adherence to follow-up care.

## 1. Introduction

Due to their increasing prevalence throughout Europe, including Romania, in terms of case numbers and illness severity, inflammatory bowel diseases (IBDs), the most common of which are Crohn’s disease (CD) and ulcerative colitis (UC), represent an important topic for research [1].

The etiology and development of IBD are influenced by a range of factors, including genetic susceptibilities, environmental influences, and gut microbiota. These factors can trigger both innate and adaptive immune responses, compromising the intestinal barrier and resulting in an increased inflammatory response within the gut [2]. To preserve intestinal homeostasis and restrict the entry of antigens into the mucosal immune system, intestinal epithelial cells are essential.

Genome-wide association studies have identified more than 300 loci linked to IBD, some of which are associated with the disturbance of intestinal homeostasis. However, most of the risk variants are found in noncoding regions, and their implication in the disease mechanism is poorly understood [3]. The occurrence of IBD rose sharply in the early industrialized world of the 20th century. The significance of environmental factors in the development of IBD depends on age at exposure. It is thought that exposures in early life—such as the use of antibiotics during the first year of life, contact with livestock and pets, and population density—can influence the risk of developing IBD. Later in life, environmental factors, such as diet and smoking, come to play a larger role in the development of IBD [4]. Along with these risk factors, in IBD, an atypical immune reaction to gut microflora occurs, which in turn leads to repetitive episodes of inflammation of the gastrointestinal (GI) tract [5].

The intestinal immune system and gut microorganisms have developed a mutually beneficial relationship over thousands of years of evolution. In healthy individuals, gut microbiota plays a crucial role in regulating the growth and function of innate and adaptive immune cells through interaction with pattern recognition receptors (PRRs), such as Toll-like receptors and NOD-like receptors [6]. On the other hand, the immune system influences the gut microbiota through different factors, such as IL-22 and secretory immunoglobulin A, which suppress the expansion of segmental filamentous bacteria [7,8]. In addition, the major histocompatibility complex class I-like molecule promotes the colonization of gut symbionts through Paneth cell function regulation [9].

Abnormal immune reactions in IBD clinically manifest as severe abdominal pain, diarrhea and cramping, weight loss, fever, and rectal bleeding [10,11].

IBD does not only affect the GI tract; it can also cause extra-digestive manifestations, such as musculoskeletal, ocular, and cutaneous problems [12]. All these symptoms can be accompanied by fatigue, depression, and anxiety, together significantly reducing quality of life [13]. Furthermore, IBD can be triggered by other comorbid conditions that can affect disease progression and the therapeutic approach. These include (i) liver diseases, such as primary sclerosing cholangitis and the global alteration of liver function; (ii) gastrointestinal comorbidities, such as infection by *Helicobacter pylori* and gluten-sensitive enteropathy; (iii) biliary and pancreatic diseases, such as cholelithiasis; (iv) obesity; (v) cardiovascular comorbidities; and (vi) psychiatric comorbidities [14,15]. In the context of IBD, the involvement of the gut–brain axis has been intensely studied in the last decade. The brain and the GI tract are crucial sensory organs, and their two-way interaction governs essential physiological and homeostatic functions. An increasing number of studies indicate that this two-way communication affects both the development and progression of functional GI disorders and is significant in the treatment of central nervous system disorders [16,17].

Evidence shows that mental disorders, in particular major depressive disorder and anxiety, adversely affect a broad range of outcomes in IBD, including increased risk of disease relapse and poorer response to treatment [18]. The prevalence of symptoms of anxiety or depression is estimated to be higher in patients with active IBD (57.6% for anxiety and 38.9% for depression symptoms) than in patients with inactive disease (38.1% for anxiety symptoms and 24.2% for depression symptoms) [19]. In IBD patients, anxiety is associated with lower medication adherence and increased risk for surgery [20]. Moreover, associations between depression and increased risk of disease relapse and poorer response to treatment in IBD have been reported [21,22]. The comorbidities and links between gastrointestinal diseases and mental disorders are well recognized [23,24,25]. It is claimed that the high rates of depression can be attributed to the symptom burden and psychological stress related to the unpredictable and uncontrollable aspects of IBD [26]. However, depression may also be an extraintestinal manifestation of IBD resulting from inflammatory activity. It has been hypothesized that excessive inflammation may be associated not only with depressive symptoms but also with the worsening of gastrointestinal symptoms that are intensified by psychological distress [27]. Similarly, psychiatric treatment contributes to psychological well-being and quality of life while also helping to reduce IBD activity and the severity of gastrointestinal symptoms [28].

It is not clear whether mental health issues contribute to IBD or if IBD causes them. Some studies have suggested that psychiatric comorbidities are risk factors for the onset of IBD since psychiatric symptoms can occur before the IBD diagnosis [29]. However, the high frequency of anxiety and depression reported after IBD diagnosis suggests that they could be consequences, rather than causes, of the disease.

In this context, clinical studies addressing the molecular fingerprint of IBD and the associated systemic/local inflammation, in connection with the presence of stress, anxiety, and depression, could bring new insights into IBD pathophysiology and provide candidate targets for combined therapy addressing both IBD and mental diseases.

MicroRNAs (miRNAs) are small, non-coding single-stranded RNA sequences that are secreted into the bloodstream. Numerous studies have reported them to be promising diagnostic, prognostic, and predictive biomarkers for a range of diseases, including cancer [30] and gastrointestinal diseases [31], as well as psychiatric disorders [32]. They exhibit remarkable stability in the blood and thus hold great potential as a less invasive alternative to tissue biopsies. MiRNAs can be found in all types of bio-fluids, including urine, saliva, milk, and cerebrospinal fluid [33]. Given that they possess several features that render them appealing targets for biomedical research, along with the challenges in acquiring human tissue samples, research on circulating miRNA continues to grow.

In this study, we sought to identify miRNA-based patterns associated with different disease conditions. We considered patients with IBD with and without symptoms of stress, anxiety, and depression and patients with psychiatric symptoms in the absence of IBD. We compared their miRNA profiles with those of non-affected controls to identify both specific and shared molecular signatures. We also wanted to pinpoint specific miRNAs able to discriminate between IBD patients with and without psychiatric symptoms. These signatures might offer molecular insights for tracking disease activity, particularly in IBD patients experiencing psychiatric symptoms that may hinder adherence to follow-up care.

With these aims, the levels of 179 miRNAs included in validated arrays for circulating miRNAs were evaluated in plasma from 20 IBD patients with no psychiatric symptoms, 19 IBD patients with symptoms of stress, anxiety and depression, 20 patients with a main diagnosis of recurrent depressive disorder, and a control group of 22 individuals with neither IBD nor psychiatric disorders.

## 2. Results

A total of 81 individuals (55.6% female and 44.4% male, mean age 40.86 ± 12.80 years) were involved in this study and were stratified into four groups based on their IBD diagnosis and psychiatric evaluation: (i) IBD (patients with IBD with no psychiatric symptoms); (ii) IBD + SAD (patients with IBD presenting with stress, and/or anxiety, and/or depressive symptoms), (iii) SAD (patients with a main diagnosis of recurrent depressive disorder); and (iv) CTRL (individuals without IBD and without psychiatric disorders). The sociodemographic and psychological characteristics of the four groups are reported in Table 1.

**Figure 1:**
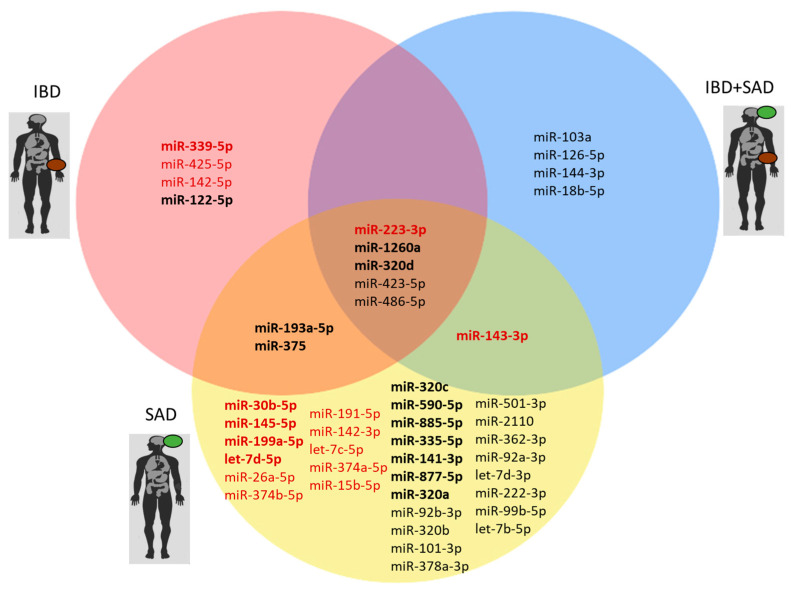
miRNAs differentially expressed in the following comparisons: IBD vs. CTRL (pink circle), IBD + SAD vs. CTRL (blue circle), and SAD vs. CTRL (yellow circle). The analysis was performed using Kruskal–Wallis tests, followed by pairwise comparisons using Dunn’s test. The red and black fonts refer to the more abundant and less abundant miRNAs in the disease condition, respectively. The miRNAs showing a FR ≥ 2 and FR ≤ −2 are reported in bold.

Among the 179 analyzed miRNAs, 46 were found differentially modified (FR ≥ 1.5 and FR ≤ −1.5, *p* < 0.05) among the three patient groups in comparison with the CTRL group. The results are shown in Figure 1.

Of the 46 miRNAs with altered levels, 30 were identified as specifically associated with the SAD phenotype, whereas 4 miRNAs were found to be specifically associated with IBD (miR-339-5p, miR-425-5p, miR-142-5p, and miR-122-5p), and another 4 with IBD + SAD (miR-103a, miR-126-5p, miR-144-3p, and miR-18b-5p) (Supplementary Table S1).

Eight miRNAs constituted a common signature of the investigated disease conditions (IBD, SAD, and IBD + SAD). In particular, miR-223-3p, miR-1260a, miR-320d, miR-423-5p, and miR-486-5p displayed statistically significant differential levels in all the comparisons vs. CTRL. miR-143-3p was also up-regulated both in IBD + SAD vs. CTRL and in SAD vs. CTRL. When IBD vs. CTRL and SAD vs. CTRL were compared, a common downregulation of two other miRNAs (miR-193a-5p and miR-375) was observed.

**Figure 2:**
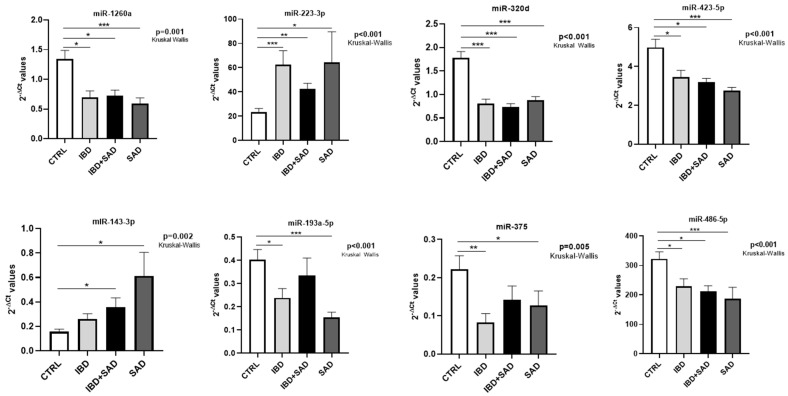
miRNAs that constitute a common signature of the three investigated disease conditions compared to the CTRL group. The bars represent the mean of the 2^−ΔCt^ values, and error bars represent the standard error. Statistical significance among the four groups was determined using Kruskal–Wallis tests, followed by pairwise comparisons using Dunn’s test. * *p* ≤ 0.05, ** *p* ≤ 0.01, and *** *p* ≤ 0.001.

To facilitate understanding of the expression trends of these eight miRNAs and the significance among the groups, a graphical representation is presented in Figure 2.

The results concerning the miRNAs that we identified as having different levels in at least two of the comparisons (SAD vs. CTRL, IBD vs. CTRL, and IBD + SAD vs. CTRL) were compared with evidence from the literature. These data are reported in Table 2. To our knowledge, no study to date has investigated the miRNA profile of patients presenting with both IBD and SAD, either in blood or in colonic tissue.

**Table 2:** Comparison of our results with those found in the literature.

miRNA	Our Results (Plasma)	Author, Year	Biological Sample	Evidence
miR-223-3p	↑ IBD vs. CTRL FR = 2.67 *p* = 0.001 ↑ IBD + SAD vs. CTRL FR = 1.81 *p* = 0.002 ↑ SAD vs. CTRL FR = 2.74 *p* = 0.027	Zhang J et al., 2023 [34]	Serum Intestinal mucosa Feces	↑ CD vs. CTRL
		Camkurt MH et al., 2015 [35]	Plasma	↑ MDD vs. CTRL
miR-1260a	↓ IBD vs. CTRL FR = −1.93 *p* = 0.012 ↓ IBD + SAD vs. CTRL FR = −1.87 *p* = 0.029 ↓ SAD vs. CTRL FR = −2.27 *p* = 0.001	No data available		
miR-320d	↓ IBD vs. CTRL FR = −2.19 *p* < 0.001 ↓ IBD + SAD vs. CTRL FR = −2.41 *p* < 0.001 ↓ SAD vs. CTRL FR = −2.04 *p* < 0.001	Ibrahim P et al., 2024 [36]	Brain EVs	↓ MDD vs. CTRL
miR-423-5p	↓ IBD + SAD vs. CTRL FR = −1.56 *p* = 0.012 ↓ SAD vs. CTRL FR = −1.81 *p* < 0.001	Yoshino Y et al., 2021 [37]	dlPFC	↑ MDD vs. CTRL
miR-486-5p	↓ IBD + SAD vs. CTRL FR = −1.52 *p* = 0.015 ↓ SAD vs. CTRL FR = −1.73 *p* < 0.001	No data available		
miR-193a-5p	↓ IBD vs. CTRL FR = −1.69 *p* = 0.022 ↓ SAD vs. CTRL FR = −2.61 *p* < 0.001	Zhou et al., 2014 [38]	PBMCs	↓ PTSD vs. CTRL
miR-375-5p	↓ IBD vs. CTRL FR = −2.68 *p* = 0.005 ↓ SAD vs. CTRL FR = −1.75 *p* = 0.039	Peck BCE et al., 2015 [39]	Colonic mucosa	↓ IBD vs. CTRL
		Wan Y et al., 2015 [40]	CSF	↑ MDD vs. CTRL
miR-143-3p	↑ IBD + SAD vs. CTRL FR = 2.28 *p* = 0.023 ↑ SAD vs. CTRL FR = 3.91 *p* = 0.002	Li SQ et al., 2022 [41]	Serum [41]	↓ MDD in association with risk of future relapse

CSF = cerebrospinal fluid, dlPFC = dorsolateral prefrontal cortex, EVs = extracellular vesicles, MDD = major depressive disorder, PBMCs = peripheral blood mononuclear cells, ↑ = high levels, and ↓ = low levels. Pearson correlation analyses between the levels of miRNAs differentially expressed in the SAD vs. CTRL comparison. The Perceived Stress Scale (PSS), Hamilton Anxiety Scale (HAM-A), and Beck Depression Inventory II (BDI-II) scales were performed, including all the study participants. Several significant correlations were observed (Table 3).

**Table 3:** Significant correlations between the level of miRNAs associated with SAD and PSS, HAMA, and BDI scales scores. The correlation was considered significant when |Pearson correlation| > 0.3 and *p* < 0.05.

miRNA Significant in SAD	PSS (Pearson Corr; *p*-Value)	HAMA (Pearson Corr; *p*-Value)	BDI (Pearson Corr; *p*-Value)
**miR-486-5p**	−0.350; 0.001	ns	ns
**miR-320d**	ns	−0.311; 0.005	ns
**miR-26a-5p**	ns	0.364; 0.001	0.339; 0.002
**let-7b-5p**	−0.317; 0.004	−0.421; <0.001	−0.394; <0.001
**miR-590-5p**	ns	−0.332; 0.002	−0.353; 0.001
**miR-320a**	−0.358; 0.001	−0.383; <0.001	−0.377; 0.001
**miR-142-3p**	ns	ns	0.358; 0.001
**let-7d-3p**	−0.351; 0.001	−0.377; 0.001	−0.318; 0.004
**miR-30b-5p**	ns	ns	0.378; 0.001
**let-7d-5p**	ns	ns	0.312; 0.005
**miR-378a-3p**	ns	ns	0.326; 0.003
**miR-501-3p**	−0.324; 0.003	ns	ns
**miR-199a-5p**	ns	ns	0.446; <0.001
**miR-92a-3p**	−0.313; 0.004	ns	ns
**miR-374a-5p**	ns	ns	0.384; <0.001

ns = not significant.

**Figure 3:**
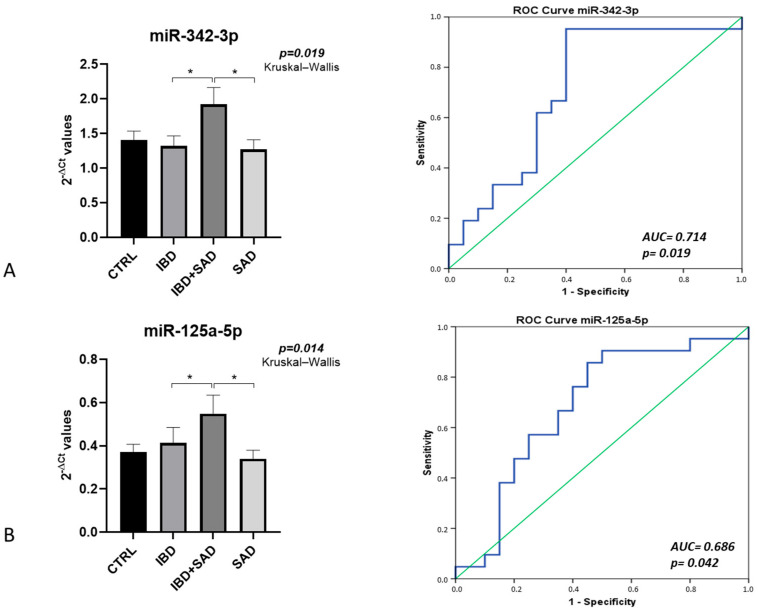
miRNAs whose levels can differentiate between the two IBD phenotypes (IBD vs. IBD + SAD). (**A**) miR-342-3p and (**B**) miR-125a-5p. In the right-hand panel, the bars represent the mean of the 2^−ΔCt^ values, and error bars represent the standard error. Statistical significance among the four groups was determined using Kruskal–Wallis tests, followed by pairwise comparisons using Dunn’s test. * *p* ≤ 0.05. The results of the ROC analysis are reported in the left-hand panel.

To identify miRNAs able to discriminate between the two IBD phenotypes (i.e., IBD vs. IBD + SAD), those with differences that were significant in the Kruskal–Wallis analysis, followed by pairwise comparisons using Dunn’s test (IBD vs. IBD + SAD), were selected for receiver operating characteristic (ROC) analysis. We found that miR-342-3p and miR-125a-5p levels were higher in the IBD + SAD group. ROC analysis of miR-342-3p and miR-125a-5p indicated that their levels could discriminate quite well between IBD and IBD + SAD, with an AUC of 0.714 (*p* = 0.019) and an AUC of 0.686 (*p* = 0.042), respectively (Figure 3).

## 3. Discussion

The objectives of this study were to find miRNA-based patterns associated with IBD, SAD, and IBD + SAD by comparing their miRNAs profile with non-affected controls in order to identify both specific and shared molecular signatures and to pinpoint specific miRNAs able to discriminate between IBD and IBD + SAD. Most studies on the expression of miRNAs in IBD have been performed with colonic tissue, with a limited number of studies that quantitatively evaluated circulating miRNA using microarrays and qRT-PCR [31].

In this study, we used qRT-PCR to assess the expression levels of 179 miRNAs in the plasma of IBD patients, IBD patients showing stress, anxiety, and depression symptoms, individuals primarily diagnosed with recurrent depressive disorder, and a control group without IBD or psychiatric disorders.

The results identified 4 miRNAs specifically associated with IBD and more than 20 miRNAs specifically related to recurrent depressive disorder, with a number of miRNAs significantly correlating with the scores of scales for stress, anxiety, and depression. In addition, we found two miRNAs (miR-342-3p and miR-125a-5p) to be potential candidates for discriminating between IBD and IBD + SAD phenotypes.

Notably, five miRNAs (miR-223-3p, miR-1260a, miR-320d, miR-423-5p, and miR-486-5p) were differentially expressed in all the comparisons. This suggests that they could represent markers of the complex phenotype that combines IBD and stress, anxiety, and depression.

To our knowledge, this is the first study to investigate the circulating miRNAs profile and considers the complex phenotype represented by IBD + SAD; hence, our findings can only partially be compared with available data from the literature.

MiR-223-3p, the mature miRNA product of the *MIR223* gene located on chromosome Xq12, plays a significant role in the differentiation and activation of cells of the immune system, and it has been reported to be involved in the development of different types of cancers, including colorectal cancer [42], which in some cases can be a complication of IBD. Being able to regulate the inflammatory process, it is highly relevant in the context of IBD [43]. The increased levels of miR-223-3p, seen in all the comparisons we performed, is in line with the results observed in IBD patients in a study conducted by Zhang and colleagues. They described higher levels of this miRNA in the serum, as well as in the intestinal mucosa and feces, of patients with CD compared to non-affected controls [34]. Furthermore, this miRNA was reported by Camkurt and colleagues to be more highly expressed in the plasma of patients with major depressive disorder [44].

Our study also revealed that miR-1260a and miR-320d were expressed at low levels in all three groups of patients compared to controls. MiR-1260a is located on the 14q24.3 chromosome region, whereas miR-320d is located on chromosome 13. Both miRNAs were reported to be involved in different types of malignancy [45]. MiR-320d has been more extensively investigated in the gut, as it may inhibit the malignant phenotype of EGFR-positive colorectal cancer (CRC) [46] and has been indicated as a putative blood biomarker for the early diagnosis of CRC [47]. Although no evidence is available in the literature regarding miR-1260a in the context of IBD and psychiatric disorders, one study indicated, in line with our results, lower levels of miR-320d in extracellular vesicles from post-mortem brains of depressed patients [36].

MiR-423-5p, miR-486-5p, miR-193a-5p, and miR-375-5p were all less abundant in the following comparisons: IBD vs. controls and SAD vs. controls. The pro-inflammatory miR-423-5p directly targets claudin-5, which is essential for maintaining the normal properties of the intestinal barrier and which, through the IL-21/miR-423-5p/CLDN5 pathway, may influence the development of IBD [48]. MiR-486-5p, miR-193a-5p, and miR-375-5p have been reported to exert a tumor suppression function in a variety of cancers, with gastric cancer being one of them [49,50,51].

Regarding the involvement of these four miRNAs in IBD and psychiatric conditions, for miR-423-5p, the only available study indicated an opposite trend in the synaptic fraction derived from the dorsolateral prefrontal cortex of patients with major depressive disorder [37]. Concerning miR-486-5p, no previous study was found. MiR-193a-5p was, instead, consistently less abundant in the peripheral blood mononuclear cells of military personnel who developed post-traumatic stress disorder [38]. Also, levels of miR-375-5p were lower in the colon tissue of patients with CD but higher in the cerebrospinal fluid of depressed patients [40,52]. Finally, regarding miR-143-3p, which we observed to be more abundant in both IBD + SAD and SAD compared to controls, lower levels were reported to be nominally associated with the risk of future relapse in patients with major depressive disorder [41].

The second aim of this study was to pinpoint specific miRNAs able to discriminate between IBD patients with and without psychiatric symptoms. MiR-342-3p and miR-125a-5p were identified as biomarkers of IBD + SAD, and both were upregulated in IBD patients with psychiatric symptoms compared to those without. Of note, both miRNAs have been reported to be involved in depression and stress. MiR-342-3p is located on the 14q32.2 chromosome region and plays a role in promoting inflammation, particularly neuroinflammation. Indeed, it has been described as a crucial mediator of TNF-α-mediated microglia activation in vitro [53] and in vivo [54]. In this last study, conducted with rats, miR-342-3p levels positively correlated with depressive-like behaviors and were significantly impacted by stress exposure. This miRNA was also indicated by a subsequent investigation to be upregulated in the blood of patients with major depressive disorder where, in addition, its expression positively correlated with TNF-α concentrations [55]. MiR-125a-5p is located on the 19q13.41 chromosome region. Upregulation of this miRNA has been described both in patients with major depressive disorder [40,56] and in those with bipolar disorder [56], although the underlying mechanisms remain unclear.

Although our findings provide, for the first time, insight regarding the circulating miRNA profile of the complex phenotypes of IBD in conjunction with stress, anxiety, and depression, this study has limitations: (i) the relatively small cohort of IBD patients did not allow statistical analysis stratifying UC and CD patients and (ii) although qRT-PCR is a common method for analyzing miRNA levels, plasma miRNAs are characterized by low abundance and high susceptibility to degradation. Thus, the results need to be confirmed with more sensitive techniques, such as digital PCR.

## 4. Materials and Methods

### 4.1. Sample Collection

In this study, 39 patients with IBD were enrolled at the Fundeni Clinical Institute of Bucharest, Romania between November2021 and November 2024. The diagnosis was established according to the guidelines from the European Crohn’s and Colitis Organization. Sixteen patients were diagnosed with UC and 23 with CD (Table 4). All the patients agreed to undergo a psychiatric consultation and were screened using the following scales: the Hamilton Anxiety Scale (HAM-A), the Becks Depression Inventory (BDI), and the Perceived Stress Scale (PSS). After psychiatric evaluation, the IBD patients were stratified in two groups: IBD (patients with IBD with no psychiatric symptoms) and IBD + SAD (patients with IBD presenting with stress, and/or anxiety, and/or depressive symptoms, according to psychiatric evaluation and the results of the HAMA-A, PSS, and BDI scales).

Twenty patients, with a main diagnosis of recurrent depressive disorder (SAD), who addressed the psychiatric service offered by the Nutrimed Clinic, Bucharest, Romania, were also enrolled and were screened using the same scales mentioned above. Additionally, another group of individuals without IBD and psychiatric symptoms (according to the results of the HAMA-A, PSS, and BDI scales) were involved in this study (CTRL). The following exclusion criteria were applied for the CTRL group: (i) the presence of digestive symptoms, (ii) current or previous nonsteroidal anti-inflammatory treatment in the past 3 months, and (iii) current or previous anticoagulant/antiplatelet treatment in the past 3 months. All patients and controls were of Romanian origin, aged over 18 years, and none reported other pathologies with an immune background. For each participant, four ml of peripheral venous blood was collected in anticoagulant-free tubes. The tubes were kept at room temperature for a maximum of 1 h and were subjected to two-step centrifugation: 1900× *g* for 10 min at 4 °C to isolate the plasma, followed by 16,000× *g* for 10 min at 4 °C to remove the presence of any cellular debris. The plasma was stored at −80 °C until miRNA isolation. This study was conducted in accordance with the Declaration of Helsinki and was approved by the local ethical committees of the Fundeni Clinical Institute (Nr. 50290-11.09.2019), Nutrimed Clinic (Nr. 1-15.04.2023), and Victor Babes National Institute of Pathology (Nr. 83-28.07.2020). All participants provided written informed consent before being enrolled in this study.

### 4.2. Scale Description

To evaluate the symptoms of stress, anxiety, and depression, we took advantage of the following scales: the Perceived Stress Scale (PSS) [57] was used to measure stress levels (0–13 = low stress, 14–26 = moderate stress, and 27–40 = high stress); the Hamilton Anxiety Scale (HAM-A) [58] was used to evaluate the severity of anxiety symptoms (0–17 = mild, 18–24 = mild to moderate, and 25–30 = moderate to severe); and the Beck Depression Inventory II (BDI-II) [59], a self-report inventory, was used to establish the severity of depressive symptoms (0–10 = normal, 11–16 = mild mood disturbance, 17–20 = borderline clinical depression, 21–30 = moderate depression, 31–40 = severe depression, and over 40 = extreme depression) [59].

### 4.3. miRNA Expression Analysis

The RNA was isolated from 200 µL of plasma using the miRNeasy Serum/Plasma Mini Kit (Qiagen, Hilden, Germany), according to the manufacturer’s instructions, and was eluted in 20 µL of nuclease-free water. Six µL of RNA was used for reverse transcription with the miRCURY LNA RT Kit (Qiagen, Hilden, Germany). The expression of 179 miRNAs (included in the validated array for circulating miRNAs) was evaluated using the Human serum/plasma focus, MIRCURY LNA miRNA Focus PCR panel YAHS-406Z (Qiagen, Hilden, Germany) using qRT-PCR. The SYBR Green chemistry on the ABI-7500 fast instrument (Applied Biosystems, Waltham, MA, USA) was used. The Ct values were normalized based on the global normalization strategy for microRNAs, considering the 127 most highly expressed miRNAs. The miRNA expression data are shown as fold regulation (FR) and as 2^−ΔCt^ values.

### 4.4. Statistical Analysis

The Shapiro–Wilk test was used to determine the miRNA expression normality distribution. Since the test revealed that the data were not normally distributed (*p*  <  0.05), statistical analysis of miRNA expression among the four groups was conducted using the Kruskal–Wallis test, followed by post-hoc pairwise comparisons with Dunn’s test and Bonferroni correction. miRNA level changes were considered significant when the *p* value was <0.05 and the FR > 1.5 or FR < −1.5. Correlations between miRNA levels and scale scores were calculated using the Pearson coefficient (r) and were considered significant at *p* ≤ 0.05. The receiver operating characteristic (ROC) curve was created, and the area under the curve (AUC) was calculated to assess the potential value of the selected miRNAs to discriminate between the two IBD phenotypes.

Statistical analysis was performed using the Statistical Package for Social Science (SPSS version 20.0), whereas graphical representations were created with GraphPad Prism 8.4.

## 5. Conclusions

This study provides new information in the field of IBD. For the first time, an association between miRNAs and psychiatric symptoms in IBD patients has been identified. In particular, we identified a pattern of miRNAs specific to the complex phenotype composed of IBD and stress, anxiety, and depression and two miRNAs able to discriminate between IBD with and without psychiatric symptoms. These specific miRNA signatures could be useful for monitoring disease activity, especially in IBD patients struggling with psychiatric symptoms that can negatively impact adherence to follow-up care.

## Figures and Tables

**Table 1:** Features of the four groups considered in this study.

	IBD (N = 20)	IBD + SAD (N = 19)	SAD (N = 20)	CTRL (N = 22)
**Sex (N, %)**	F (10; 50.0%) M (10; 50.0%)	F (11; 57.9%) M (8; 42.1%)	F (13; 65.0%) M (7; 35.0%)	F (11; 50.0%) M (11; 50.0%)
**Age (mean ± SD)**	38.85 ± 14.80 (22.00–70.00)	42.57 ± 16.22 (21.00–74.00)	37.95 ± 11.91 (23.00–63.00)	43.86 ± 6.99 (25.00–53.00)
**HAM-A (N, %)**	Mild (20; 100%) Mild to moderate (0; 0%) Moderate to severe (0; 0%)	Mild (13; 68.42%) Mild to moderate (3; 15.79%) Moderate to severe (3; 15.79%)	Mild (11; 55%) Mild to moderate (3; 15%) Moderate to severe (6; 30%)	Mild (22; 100%) Mild to moderate (0; 0%) Moderate to severe (0; 0%)
**BDI (N, %)**	Normal (19; 95%) Mild mood disturbance (1; 5%) Borderline (0; 0%) Moderate (0; 0%) Severe (0; 0%) Extreme (0; 0%)	Normal (12; 63.17%) Mild mood disturbance (3; 15.79%) Borderline (1; 5.26%) Moderate (1; 5.26%) Severe (2; 10.52%) Extreme (0; 0%)	Normal (1; 5%) Mild mood disturbance (5; 25%) Borderline (2; 10%) Moderate (10; 50%) Severe (1; 5%) Extreme (1; 5%)	Normal (22; 100%) Mild mood disturbance (0; 0%) Borderline (0; 0%) Moderate (0; 0%) Severe (0; 0%) Extreme (0; 0%)
**PSS (N, %)**	Low stress (18; 90%) Moderate stress (2; 10%) High stress (0; 0%)	Low stress (2; 10.53%) Moderate stress (13; 68.42%) High stress (4; 21.05%)	Low stress (4; 20%) Moderate stress (10; 50%) High stress (6; 30%)	Low stress (11; 50%) Moderate stress (11; 50%) High stress (0; 0%)

Legend: IBD, patients with IBD with no psychiatric symptoms; IBD + SAD, patients with IBD presenting with stress, and/or anxiety, and/or depressive symptoms; SAD, patients with a main diagnosis of recurrent depressive disorder; CTRL, individuals without IBD and without psychiatric disorders. HAM-A, Hamilton Anxiety Scale; BDI, Becks Depression Inventory; PSS, Perceived Stress Scale.

**Table 4:** Clinical and demographic characteristics of the IBD patients.

IBD Tot (N = 39)	
**Sex (N, %)**	F (21; 53.8%) M (18; 46.2%)
**Age (mean ± SD)**	40.66 ± 15.42
**Disease type**	UC (16; 41.0%) CD (23; 59.0%)
**Disease duration (mean ± SD)**	9.04 ± 7.23
**Disease state (N, %)**	Remission (27; 69.2%); Active (12; 30.8%)
**Treatment type (N, %)**	Biological (35; 89.7%); Other (4; 10.3%)
**UC Montreal classification**	E1: Proctitis (N = 0) E2: Left-sided colitis (N = 9) E3: Extensive colitis (N = 7)
**Location of Crohn’s**	Location of Crohn’s (N = 23) L1: Ileal (N = 3) L2: Colonic (N = 7) L3: Ileocolonic (N = 13) Upper GI involvement (N = 1)
**Crohn’s behavior**	B1: Inflammatory (N = 10) B2: Stricturing (N = 9) B3: Penetrating (N = 4) Perianal involvement (N = 6)
**CRP, mg/L (mean ± SD) (min–max)**	9.20 ± 11.70 (0.50–45.30)
**Fecal calprotectin, μg/g (mean ± SD) (min–max)**	689.51 ± 974.42 (5.00–4223.00)

## Data Availability

Raw data can be obtained from the corresponding author upon reasonable request.

## Supplementary Materials

The following supporting information can be downloaded at: https://www.mdpi.com/article/10.3390/ijms26157321/s1.

## Abbreviations

The following abbreviations are used in this manuscript:

IBD	Inflammatory Bowel Disease
CD	Crohn’s Disease
UC	Ulcerative Colitis
GI	Gastrointestinal
SAD	Stress, Anxiety, and Depression
HAM-A	Hamilton Anxiety Scale
BDI	Becks Depression Inventory
PSS	Perceived Stress Scale
