# Supplementary material for: Circulating miRNA Profile in Inflammatory Bowel Disease Patients with Stress, Anxiety, and Depression
Source: Int J Mol Sci. 2025 Jul 29;26(15):7321. doi: 10.3390/ijms26157321 (PMC12347236; doi:10.3390/ijms26157321)
Supplement: Supplementary file 1 [file ijms-26-07321-s001.zip › ijms-3740735-supplementary.pdf]

Table S1. MiRNAs presenting significant altered levels.

**miRNA**	**IBD *vs* CTRL**		**IBD+SAD *vs* CTRL**		**SAD *vs* CTRL**	
	FR	p-value	FR	p-value	FR	p-value
**miR-374b-5p**					1,81	<0,001
**miR-145-5p**					2,33	0,001
**miR-486-5p**		0,043	-1,52	0,015	-1,73	<0,001
**miR-26a-5p**					1,72	<0,001
**miR-143-3p**			2,28	0,023	3,91	0,002
**miR-222-3p**					-1,55	<0,001
**miR-375**	-2,68	0,005			-1,75	0,039
**let-7b-5p**				0,007	-2,01	<0,001
**miR-99b-5p**					-1,57	0,042
**miR-320b**					-1,84	<0,001
**miR-590-5p**					-2,23	<0,001
**miR-191-5p**					1,60	0,001
**miR-122-5p**	-3,77	0,009				
**miR-423-5p**		0,015	-1,56	0,012	-1,81	<0,001
**miR-101-3p**					-1,50	0,006
**miR-320a**		0,027		0,012	-2,05	<0,001
**miR-223-3p**	2,67	<0,001	1,81	0,002	2,74	0,027
**miR-103a**			-1,67	0,029		
**miR-142-3p**					1,74	<0,001
**let-7d-3p**					-1,54	0,003
**miR-126-5p**			-1,55	0,005		
**miR-144-3p**			-1,51	0,034		
**miR-18b-5p**			-1,80	0,001		
**miR-335-5p**					-2,02	0,006
**miR-320d**	-2,19	<0,001	-2,41	<0,001	-2,04	<0,001
**miR-30b-5p**					2,07	<0,001
**miR-92b-3p**					-1,84	0,02
**miR-141-3p**					-2,25	0,01
**let-7d-5p**					2,02	<0,001
**miR-320c**					-2,14	<0,001
**let-7c-5p**					1,85	0,001
**miR-1260a**	-1,93	0,012	-1,87	0,029	-2,27	0,001
**miR-142-5p**	1,54	0,027				
**miR-362-3p**					-1,86	0,012
**miR-339-5p**	2,19	0,003				
**miR-378a-3p**				0,022	-1,52	0,002
**miR-425-5p**	1,51	0,007				
**miR-193a-5p**	-1,69	0,022			-2,61	<0,001
**miR-885-5p**					-2,04	0,014
**miR-501-3p**					-1,78	0,002
**miR-2110**					-1,81	0,047
**miR-199a-5p**					2,43	0,003
**miR-92a-3p**					-1,61	0,002
**miR-374a-5p**					1,93	<0,001
**miR-877-5p**					-2,56	<0,001
**miR-15b-5p**					1,67	0,001
